# Aggregate size distribution in a biochar-amended tropical Ultisol under conventional hand-hoe tillage

**DOI:** 10.1016/j.still.2016.08.012

**Published:** 2017-01

**Authors:** Bernard Fungo, Johannes Lehmann, Karsten Kalbitz, Margaret Thionģo, Irene Okeyo, Moses Tenywa, Henry Neufeldt

**Affiliations:** a CGIAR Research Program on Climate Change, Agriculture and Food Security (CCAFS), World Agroforestry Center (ICRAF), P. O. Box 30667, UN Avenue-Gigiri, Nairobi, Kenya; b Institute for Biodiversity and Ecosystem Dynamics (IBED), Faculty of Science, University of Amsterdam, Science Park 904, 1098 XH Amsterdam, The Netherlands; c National Agricultural Research Organization (NARO), P. O. Box 1752, Kampala, Uganda; d Soil and Crop Sciences, Cornell University, Bradfield Hall, Ithaca, NY 14853, USA; e College of Agricultural and Environmental Sciences, Makerere University, P. O. Box 7062, Kampala, Uganda; f Soil Resources and Land Use, Institute of Soil Science and Site Ecology, Dresden University of Technology, Pienner Strasse 19, 01737 Tharandt, Germany

**Keywords:** Biochar, Soil aggregation, Soil organic carbon, Soil respiration, Ultisol, Hand-hoe tillage

## Abstract

Biochar (or pyrogenic organic matter) is increasingly proposed as a soil amendment for improving fertility, carbon sequestration and reduction of greenhouse gas emissions. However, little is known about its effects on aggregation, an important indicator of soil quality and functioning. The aim of this study was to assess the effect of Eucalyptus wood biochar (B, pyrolyzed at 550 °C, at 0 or 2.5 t ha^−1^), green manure (T, from *Tithonia diversifolia* at 0, 2.5 or 5.0 t ha^−1^) and mineral nitrogen (U, urea, at 0, or 120 kg N ha^−1^) on soil respiration, aggregate size distribution and SOC in these aggregate size fractions in a 2-year field experiment on a low-fertility Ultisol in western Kenya under conventional hand-hoe tillage. Air-dry 2-mm sieved soils were divided into four fractions by wet sieving: Large Macro-aggregates (LM; >1000 μm); Small Macro-aggregates (SM, 250–1000 μm); Micro-aggregates (M, 250–53 μm) and Silt + Clay (S + C, < 53 μm). We found that biochar alone did not affect a mean weight diameter (MWD) but combined application with either *T. diversifolia* (BT) or urea (BU) increased MWD by 34 ± 5.2 μm (8%) and 55 ± 5.4 μm (13%), respectively, compared to the control (*P* *=* *0.023*; n = 36). The B + T + U combination increased the proportion of the LM and SM by 7.0 ± 0.8%, but reduced the S + C fraction by 5.2 ± 0.23%. SOC was 30%, 25% and 23% in S + C, M and LM/SM fractions, and increased by 9.6 ± 1.0, 5.7 ± 0.8, 6.3 ± 1.1 and 4.2 ± 0.9 g kg^−1^ for LM, SM, M and S + C, respectively. MWD was not related to either soil respiration or soil moisture but decreased with higher SOC (*R^2^* *=* *0.37, P* *=* *0.014, n* *=* *26*) and increased with greater biomass production (*R^2^* *=* *0.11, P* *=* *0.045, n* *=* *33*). Our data suggest that within the timeframe of the study, biochar is stored predominantly as free particulate OC in the silt and clay fraction and promoted a movement of native SOC from larger-size aggregates to the smaller-sized fraction in the short-term (2 years).

## Highlights

• Biochar does not affect soil aggregation on the humid Ultisol within two years.
• Biochar, when applied with *T. diversifolia,* increased soil aggregation.
• Mineral fertilizer reduced macro-aggregate stability in the short-term.
• SOC increases in the micro-aggregates but reduces in macro-aggregates.

## 1 Introduction

Biochar (pyrogenic organic matter) has shown promise for contributing to the triple benefit of improving soil productivity (Biederman and Harpole, 2013, Qian et al., 2015), sequestering soil carbon (Lehmann, 2007, Schneider et al., 2011, Lorenz and Lal, 2014) and reducing an emission of greenhouse gasses (i.e. CO_2_, CH_4_ and N_2_O) in agricultural soils (Cayuela et al., 2013, Fungo et al., 2014). According to Woolf et al. (2010), sustainable global implementation of biochar projects can potentially off-set 12% (1.8 Pg CO_2_-C_e_ per year) of current anthropogenic CO_2_-C equivalent emissions. However, the rate and scale of soil organic matter (SOM) turnover following biochar amendment depends on complex associations among biochar as well as soil properties (pH, native SOM, texture, mineralogy), agro-ecological conditions (precipitation and temperature), and management interventions such as use of manure and mineral fertilizers, tillage and irrigation.

Soil aggregation is a good indicator of soil quality because it mediates microbial feedbacks of C and N cycling in soils (Kapkiyai et al., 1999, Jimenez et al., 2011, Demisie et al., 2014). Biochar incorporation into soil can improve soil aggregate stability (Liu et al., 2014, Zhang et al., 2015, Obia et al., 2016) by increasing exchangeable cation status of the soil, such as calcium (Enders et al., 2012, Jien and Wang, 2013), thereby inhibiting clay dispersion and associated disruption of soil aggregates. Biochar can also affect aggregation by the replacement of Na^+^ and Mg^2+^ in clay and aggregates through adsorption on its surfaces (Kwon and Pignatello, 2005). Under acidic environments such as those in highly weathered soils of the humid tropics, the hydroxyl and carboxylic groups on the oxidized biochar surface could also adsorb clay particles to increase macro-aggregate formation (Jien and Wang, 2013). However, the location of SOC within the aggregates and its chemical characteristics, which affect the rate of its decomposition (Balesdent et al., 1998, Christensen, 1996, Luo et al., 2014) and thus is sequestration potential, have not received much attention.

The effect of biochar on soil aggregation is disputed (c.f. Busscher et al., 2010, Peng et al., 2011, Zhang et al., 2015). Whereas an increase in soil aggregate sizes as a result of an increase in SOC when synthetic fertilizers are applied to the soil has been widely reported (Halvorson et al., 1999, Plaza-Bonilla et al., 2012), some evidence of the reverse trend has also been observed (Sainju et al., 2003, Khan et al., 2007, Le Guillou and Angers, 2011, Plaza-Bonilla et al., 2012). Biochar is expected to increase aggregation because it can act as a nucleus of aggregation, similar to other particulate organic matter or microorganisms, especially because biochar increases microbial biomass (Lehmann et al., 2011). Furthermore, increased OM input by roots and microbial mucilage following biochar amendment would increase aggregation (Abiven et al., 2015). Hence, it is unclear how N fertilizers in combination with biochar can affect both soil aggregate size distribution and the resultant physical protection of SOC.

When biochar is applied with green manure as *Tithonia diversifolia*, there is likely a greater amount of microbial activity (Li et al., 2012) and concomitant production of metabolites which, through a variety of bonding mechanisms, may contribute to aggregate build-up. Mechanisms of interaction between biochar and the soil matrix that may result in soil stabilization include (1) occlusion in aggregates (Bachmann et al., 2008), (2) formation of biochar-cation complexes (interactions with polyvalent cations of soil minerals), or (3) interactions via polyvalent cations with soil mineral surfaces (OM-mineral associations) (von Lützow et al., 2007). Thus, biochar can be a binding agent for aggregate formation and stabilization. However, our understanding of these effects on aggregation of soil remains speculative. Understanding the effect of introducing biochar in such a system will aid predicting the long-term effects of these cropping practices on soil quality and C storage.

The objectives of the study were to determine the effect of biochar on (i) size and distribution of soil aggregates, (ii) changes in the content of SOC in different soil fractions, and (iii) relationships among aggregation, SOC, soil respiration (CO_2_ emission) and biomass production under integrated soil fertility management on an Ultisol of the humid tropics. We hypothesized that under conventional hand-hoe tillage practices, (i) biochar would increase soil aggregation because over time, biochar gets more oxidized (Cheng et al., 2008), so there may be more cation bridges between clay and biochar (increasing its ability to form organo-mineral and Biochar-SOM interactions), (ii) soil aggregation increases with an increased amount of easily mineralizable organic matter inputs (such as *T. diversifolia* manures) because of the increased microbial activity and therefore mucilage, but might decrease with addition of mineral N fertilizer (such as urea) because of increased decomposition of easily mineralizable SOM, and (iii) increased soil respiration is related to SOM increases and larger aggregates.

## 2 Materials and methods

### 2.1 Study site

The field experiment was established in September 2012 at Kapsengere on the southern Nandi hills in western Kenya. The sites receive ∼2000 mm mean annual rainfall in a bimodal distribution, with two rainy seasons per year (March–July and September–January) with a mean annual temperature of 26 °C. Precipitation and air temperature were monitored throughout the experiment with the help of a weather station located near the experimental field. The soil is classified as Typic Kandiudults (USDA, 1999) developed on biotite-gneisses parent material. The natural vegetation is composed of tropical rainforest of Guineo-Congolian species. The trial was conducted for four consecutive maize rainy seasons (September 2012–August 2014).

### 2.2 Preparation of the biochar and *T. diversifolia*

The biochar was produced by chopping and grinding Eucalyptus wood so as to pass through a 2-mm sieve. The sieved material was then pyrolyzed at a ramp of 5 °C min^−1^ to a maximum temperature of 550 °C and retained for one hour before cooling to room temperature. In the laboratory, the resultant biochar was characterized for pH, surface area, CEC, elemental composition. *T. diversifolia* was prepared by cutting leaves from the field and chopping them into 50-mm lengths, air-dried and ground to pass through a 1-mm sieve before field application. The chopping and grinding were to ensure uniform application in the field and reduce effects on soil physical properties. The physical and chemical characteristics of the above materials are presented in Table 1.

### 2.3 Experimental design

The treatments were selected to represent presence and absence of biochar as well as low and high input of *Tithonia* green manure, with and without mineral nitrogen (N) fertilizer. This arrangement represented a range of conventional management practices of many small-holder farmers in integrated soil fertility management systems. Most farmers in the study area are small scale, resource poor, mixing small quantities of each of these amendments. The experiment was laid out in a randomized complete block design with three replicates. The treatments included the following: two levels of biochar (0 and 2.5 t ha^−1^); three levels of green manure applied as *T. diversifolia* (0, 2.5 and 5 t ha^−1^); and two levels of mineral N applied as Urea (0 and 120 kg N ha^−1^) (Table 2). Each treatment was established in a 2 × 2-m plot separated by a one-meter distance within and between rows. Due to the inherently low fertility of the soil, 30 kg ha^−1^ of P_2_O_5_ as TSP and 30 kg ha^−1^ of K_2_O as Muriate of Potash were applied to each plot.

### 2.4 Management of experiment

Conventional tillage, where a hand-hoe is used to mix the top 0.10–0.15 m of the soil, was used during land preparation at the start of each season, and the two weeding times during each season. Application of biochar was done once at the start of the first season on 3rd October 2012. Constant amounts (2.5 or 5 t ha^−1^, Table 2) of green manure, phosphorus (TSP) and potassium (MoP) were applied to each plot once at the start of each season. Mineral N (urea) was applied in two splits; 40% at planting and 60% 30 days after planting. The biochar, manure and mineral fertilizer were broadcast on the soil surface by hand and incorporated into the top 0.1 m soil. Two seeds of the maize cultivar HB 513 were planted at a spacing of 0.25 m within and 0.5 m between rows (40 plants per planting hole). Weeding was done at 30 and 50 days after planting then tilled with a hand-hoe to a depth of 0.1 m. Thinning was done during the first weeding to retain one plant per pocket. In total, four consecutive seasons of maize crop were harvested (3rd October 2012 through 17th August 2014).

### 2.5 Soil respiration and above ground biomass

We used data on soil respiration (CO_2_ evolution at the soil surface) and aboveground biomass. Briefly, measurements were conducted using a static closed chamber method. The chamber consisted of a PVC tube (diameter = 0.3 m; height = 0.15 m) transversely divided into two parts to make a base (0.05 m) and a cover (0.1 m). The base was driven into the soil to ∼0.02 m below the soil surface. To ensure air-tight conditions, a rubber ring was placed between the base and the cover. A photo-acoustic infrared field gas monitor (INNOVA 1402, Lumasense Technologies A/S, Ballerup, Denmark) was used to analyze the fluxes in the field. The gas monitor was connected to the chamber by two 0.7 m-long Teflon tubes as gas inlet and outlet. Inside the cuvette, air humidity and temperature were monitored by a digital thermo-hygrometer (PCE-313A, Paper-Consult Engineering Group, Meschede, Germany) attached to the cover from the outside and only the sensor reached inside the chamber through a rubber screw connector. Two chambers were set up in each plot. For each gas sampling event INNOVA recorded four measurements at 2-min intervals after closing the chamber. Flux measurements were conducted weekly except during dry periods where bi-monthly measurements were taken. Measurements were taken at a similar time during the day (9–11a.m.). Temperature ranged from 23 to 28 °C. No significant relationship between temperature and CO_2_ flux was observed.

### 2.6 Soil sampling and analysis

Composite soil samples were taken from five random locations within each plot from a 0–0.15 m depth on 17th August 2014 (24 months after biochar application). Soil cores (*d* = 50 mm, *l* = 50 mm; *v* = 100 cm^3^) were used to collect samples for bulk density determination. Approximately 200 g of the air-dry, 2-mm sieved soil samples was packed in zip-locks and taken to the laboratory for analysis.

### 2.7 Soil fractionation and chemical analysis

Particle size fractionation procedure was used to determine the mean weight diameter (MWD) as an indicator of soil aggregate distribution. Bulk soil was divided into four size fractions; (i) Large Macro-aggregates (>1000 μm, designated LM); (ii) Small Macro-aggregates (250–1000 μm, designated SM); (iii) Micro-aggregates (250–53 μm, designated M) and (iv) Silt + Clay (<53 μm, designated S + C). Four sieves corresponding to these size classes were sequentially arranged vertically. Eighty grams of an air-dried soil sample, without disturbing the aggregates was put on the first sieve of the set in a water bucket and was gently moistened for 10 min. After the soil was moistened with water, aggregates were separated by moving the sieve vertically at 30 strokes min^−1^ for 5 min. At the end of wet-sieving, all aggregate-size fractions remaining on each sieve were collected, dried at 60 °C, and the sand and aggregates were separated (Wang et al., 2012). The mean weight diameter (MWD, mm) was calculated as follows:

$$\text{MWD}=\overset{n}{\underset{i=1}{\sum }}w_{i}.\bar{x_{i}}$$

where $\bar{x}$ is the average diameter of the openings of the two consecutive sieves, and $w_{i}$ the weight ratio of aggregates remained on the ith sieve. For the determination of aggregate size distribution, the weight ratio of aggregates of each sieve to the total weight of aggregates was calculated. Then, the C and N content in the various size fractions was determined. Soil pH_water_ was determined with a glass electrode (Soil:Water = 1:5 w/v). Soil organic C and total N were measured with an Elementar Vario max CNS Analyzer (German Elementar Company, 2003). It was assumed that TOC = SOC since these acid soils have negligible amounts of inorganic carbonates.

### 2.8 Statistical analysis

The cumulative CO_2_ flux for each treatment was derived using a linear Trapezoidal rule with sampling dates as the time intervals. Changes in SOC content were calculated as the difference between values at the beginning and end of the sampling period, as well as subtracted the C addition from biochar and *T. diversifolia*. Biochar-induced differences for each treatment were calculated as the difference between the treatment value and that of the control. Treatment main effects and their interaction on MWD and C content in soil aggregates were examined using fixed effect analysis of variance (ANOVA). Post Hoc separation of means was done using Least Significant Difference (LSD) at 5%. Linear regression was used to study the relationship between MWD and SOC, aboveground biomass and soil respiration as well as that between SOC and above ground biomass (C).

## 3 Results

### 3.1 Distribution and MWD of water stable aggregates

**Fig. 1:**
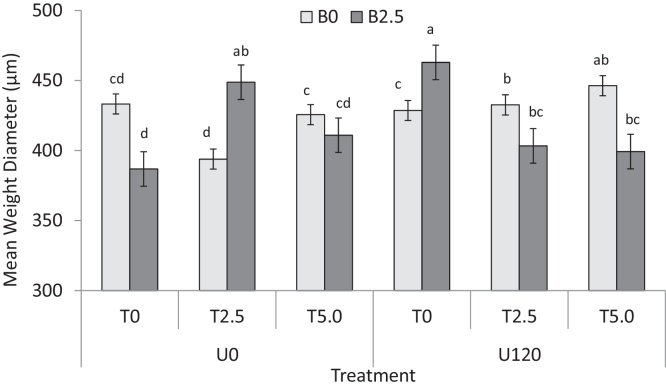
Effect of additions of biochar (B), *T. diversifolia* (T) green manure and urea (U) on mean weight diameter of soil aggregate (values indicate amendment rate in t ha^−1^). Error bars are standard error, n = 3, means with the same letter are not significantly different at p < 0.05.

The values of MWD ranged from 378 μm to 525 μm (mean ± SE = 423 ± 23) (Fig. 1). The biochar addition had no effect on MWD, but the combination of biochar with either *Tithonia* (B_2.5_ + T_2.5/5_) or urea (B_2.5_ + U_120_) significantly increased MWD by 34 ± 5.2 μm (8%) and 55 ± 5.4 μm (∼13%), respectively compared to the control. The B_2.5_ + T_2.5/5_ and B_2.5_ + U_120_ treatments were themselves not significantly different. The T_2.5/5_ + U_120_ treatment significantly increased MWD by 17 ± 4.1 μm (Fig. 1) compared to the control and the rate of *Tithonia* addition had no significant effect on MWD. The MWD of the B_2.5_ + U_120_ was comparable to that of the B_2.5_ + T_2.5_ treatment, and was significantly lower than B_2.5_ + T_5.0_. MWD was not significantly different from the control under the three-amendment mixture (P > 0.05).

**Fig. 2:**
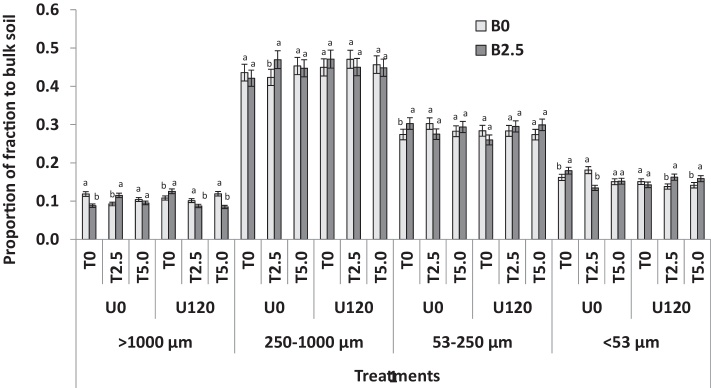
Proportion of different size aggregates in bulk soil of an Ultisol 2 years after amendment with biochar (B), T. diversifolia (T) green manure and urea (U) (values indicate amendment rate in Mg ha^−1^). Error bars are standard error, n = 3, means with the same letter are not significantly different at p < 0.05.

Table 3 shows the results of ANOVA of the main effects of each amendment as well as their interactions while Fig. 2 shows the effect of the treatments on the distribution of the different aggregate size fraction in the bulk soil. The SM dominated the size distribution (45%) followed by M fraction (29%), then S + C (15%). The LM were the least represented fraction (10%) (Table 3). Sole biochar treatment had no effect on size proportion. Sole *T. diversifolia* increased the S + C fraction by 8% (*F* *=* *3.8; P* *=* *0.030*) after two years of the field trial.

Overall, the proportion of LM increased by 53% while the S + C fraction decreased by 46% over the two years of the field experiment (Fig. 2). The proportion of the LM fraction reduced by 14% but the proportion of the M fraction did not change over the two years. The B + T and B + U treatments significantly increased the proportion of the SM fractions by 15% (Table 3). There was no significant difference in size proportion between T_2.5_ and T_5_. Sole urea additions decreased the proportion of LM but increased the S + C fraction (Fig. 2). The B + T + U mixture significantly increased the proportion of the LM by 7.0 ± 0.8%, but significantly reduced the proportion of the S + C fraction by 5.2 ± 0.23%, independent of the amount of *T. diversifolia* (Fig. 2). The S + C fraction was not affected by any of the amendments, in sole or in combined application.

### 3.2 SOC in aggregates

The S + C fraction contained the largest proportion of SOC (30%) followed by M (25 g kg^−1^). The LM and SM had a similar mean content of SOC content (23 g kg^−1^). At the end of the two years, mean SOC contents in all the soil fractions had increased by a range of 0.44–4.69 g kg^−1^ (1.92 ± 1.06 = Mean ± SE). The increase in SOC was 9.6 ± 1.0, 5.7 ± 0.8, 6.3 ± 1.1 and 4.2 ± 0.9 g kg^−1^ for LM, SM, M and S + C, respectively. The increase in SOC content in LM was significantly higher than for the SM, and the SM was not significantly different from M but significantly higher than the SOC content in the S + C fraction. Overall, biochar and *T. diversifolia* increased SOC. In the S + C fraction, urea and *T. diversifolia* together increased SOC by 2.0 ± 0.2 g kg^−1^ (∼7%) without biochar but had no effect when biochar was added.

### 3.3 MWD, SOC and soil respiration

**Fig. 3:**
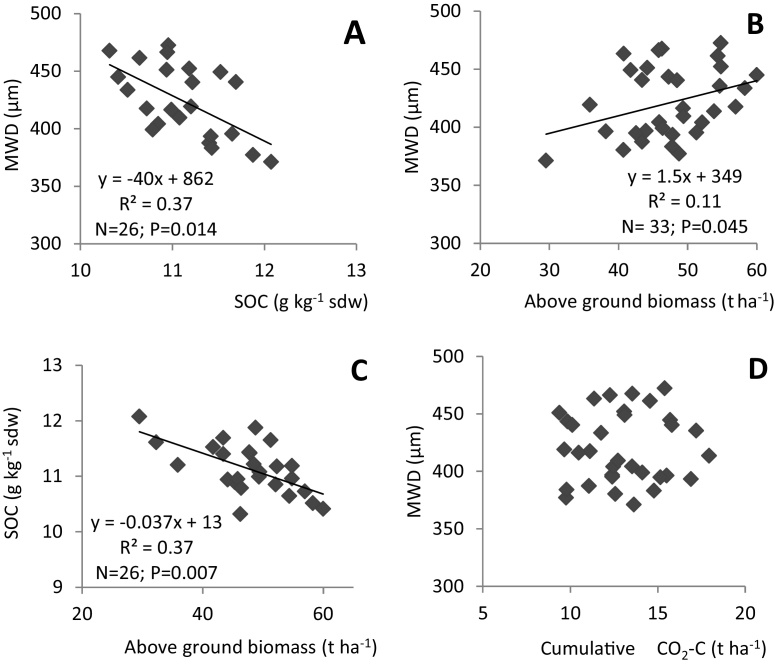
Linear relationships between MWD and SOC (A), aboveground biomass (B) and soil respiration (D) and that between SOC and above ground biomass (C).

MWD was inversely related to SOC and SOC increase explained 37% of the decrease in MWD (Fig. 3A). In addition, MWD increased with increasing aboveground biomass. The amount of increase in MWD attributed to biomass production was 11% (Fig. 3B). SOC and aboveground biomass were also inversely related (Fig. 3C). There was no significant relationship between MWD and soil respiration (CO_2_ emission) (Fig. 3D).

## 4 Discussion

### 4.1 Size and distribution of soil aggregates

Our expectation that biochar would consistently increase soil aggregate formation, was not met entirely. Biochar alone may not have increased aggregate size significantly after two years because aggregates formed in the early stages could have been broken down due to tillage at planting, and weeding. Our results are in agreement with those of others (e.g. Herath et al., 2014, Peng et al., 2016) who found no effect of biochar on micro-aggregation. However, other studies have reported increases in aggregate size (Sun and Lu, 2013, Liu et al., 2014, Abdelhafez et al., 2014). Differences in the effect have probably occurred due to time, application rate and texture of biochar used. For example, Liu et al. (2014) reported increased aggregation at 40 t ha^−1^, but not at 20 t ha^−1^. Sun and Lu (2013) also reported increased aggregation with 90 t ha^−1^ straw biochar but no difference with wood chips biochar at the similar rate.

The positive relationship between MWD and biomass growth (Fig. 3B) could be due to increased easily mineralizable C input. Ability of biochar to improve soil structure and infiltration can also increase water viscosity, thereby increasing soil aggregation (Bandyopadhyay and Lal, 2014, Regelink et al., 2015). However, this relatively weak relationship suggests either short-term build-up of unstable soil aggregates, which soon break down, or time was insufficient for a slow buildup of aggregates. It is possible that aggregation could have been limited by the type of microbially derived OM during the decomposition and degradation of *T. diversifolia* and biochar, respectively. According to Bronick and Lal (2005), easily decomposable inputs such as green manure have strong but transient effects on aggregate stability while more recalcitrant inputs such as decomposed manures would show weak but long-term effects. There is also evidence that mucilage types (or chemical saccharides compositions) and amounts secreted from different plant species, as well as soil moisture levels and soil fauna also influence soil aggregate structure (Degens and Sparling, 1996, Six et al., 2004, Bossuyt et al., 2005).

The indifference in MWD with biochar additions alone could be related again to the quantity and quality of biochar applied (texture, pH, CEC). The soil used in our study was an Ultisol, which is relatively high in 1:1 clay, low in CEC, and in base cations and we would expect such a soil to respond to biochar amendment by increasing aggregation (cf Gentile et al., 2010, Ouyang et al., 2013). According to Gentile et al. (2010), aggregates of fine-textured soils are more responsive to organic matter inputs compared to the coarse-textured ones. However, Liu et al. (2012) found increased aggregation in two silt loam soils but not in two sandy loam soils, suggesting that higher clay content would increase likelihood of aggregation. Our soil was dominated by relatively a larger particle size fraction (250–1000 μm), which could partly explain the limited response. The carboxylic and phenolics, which are the predominant functional groups responsible for surface charge in biochar, decreased with increased pyrolysis temperature (Amonette and Joseph, 2009, Keiluweit et al., 2010). The feedstock used for making the biochar as well as the relatively high pyrolysis temperature (550 °C) of our biochar could have resulted in lower surface charge as indicated by relatively low CEC (Table 1), hence low propensity for aggregation over the time period studied here.

The increase in the proportion of micro-aggregates with *T. diversifolia* and urea could be related to the increased biomass C from microbial C after decomposition of *T. diversifolia*, as well as plant root biomass. Indeed Whalen and Chang (2002) showed that the increase in SOC caused by the application of manures is a direct result of the manure composition and an indirect result of the increased crop growth and crop residue in response to the nutrient supply. During SOM decomposition by microorganisms, synthesis of hydrophilic polysaccharides promotes inter-particle cohesion through adsorption to mineral matter (Chenu, 1989, Verchot et al., 2011, Demisie et al., 2014), thus increasing soil aggregation.

### 4.2 SOC in soil aggregate

We postulated that easily mineralizable C derived from *T. diversifolia* could end up in the micro-aggregates. These micro-aggregates would later be incorporated into macro-aggregates. The build-up of micro-aggregate C observed in our study (Table 4) is in support of the “bottom-up” process of soil aggregation proposed by Verchot et al. (2011) whereby micro-aggregates form through the interaction between mineral surfaces and organic matter with little protection in early stages of micro-aggregate formation (Emerson, 1959, Tisdall and Oades, 1982, Lehmann et al., 2007). This proposition is further supported by Kinyangi et al. (2006), and Verchot et al. (2011) that aliphatic-C, which tends to form thin films on mineral surfaces and is found throughout the microstructure of the aggregates, appears to be the responsible agent for stabilization of micro-aggregates. Verchot et al. (2011), using δ^13^C, also found that the portion of the new carbon from the trees in an agroforestry fallow was sequestered in the micro-aggregate.

We did not observe a significant change in macro-aggregate C content since our biochar was predominantly fine-textured (<250 μm) compared to Herath et al. (2014) for example, who had relatively larger-sized particle sizes of biochar. On the other hand, some studies (c.f. Herath et al., 2013, Zhang et al., 2015) found higher C in macro-aggregate fractions than in the smaller ones, indicating that biochar amendments could particularly increase C storage in these larger macro-aggregate fractions as free particulate organic matter. Although air-drying soils before fractionation can have affected on aggregation to some extent (Warkentin and Maeda, 1980), we assumed that the effect, if any, was similar for all treatments.

### 4.3 Relationship between MWD and SOC, soil respiration and biomass production

The negative relationship between MWD and SOC could be explained by the fact that at this time scale, the highest values of MWD were related to the microbial activity induced by the green manure. In our case, the soil is highly weathered with free ions such as Al and Fe as well as sesquioxides, and this could significantly curtail aggregation over relatively short time scales (<10 years). Arthur et al. (2014) also observed no relationship between SOC and aggregation when several soil types were amended with 7.5 t ha^−1^ of ground rape shoot manure. In our case, the amount of manure added was less than that used by Arthur et al. (2014) and that could partly explain the absence of any significant response.

The increase in CO_2_ emission (soil respiration) is attributed to the increase in mineralizable C particularly in relatively low-C soils (Sagrilo et al., 2014). Improvement in soil aeration, following biochar addition has also been reported but the lack of a change in MWD and bulk density rules out this explanation from our study.

Increased plant growth was expected to increase aggregation via OM input in associated root biomass, but a reverse relationship was observed instead. Also, we did not observe a significant relationship between MWD and CO_2_ evolution. Mizuta et al. (2015) found that although polysaccharides such as starch and cellulose accelerated soil aggregation, the decomposition of these amendments influenced only aggregation, not aggregate stabilization. It has also been reported that the role of organic matter (aggregating or disaggregating) depended on its chemical composition and presence of other binding agents (Goldberg et al., 1990, Mizuta et al., 2015, Wu et al., 2016). The conventional hand-hoe tillage system used in this study is what is practiced by most farmers in the area and it may compromise short-term build-up of soil aggregates. Therefore, within the timeframe of this study, the aggregated distribution benefits of relatively low organic input may not be evident. Long-term trials testing various tillage practices are required to clarify the interaction between biochar and other amendments on aggregation and stabilization of soil aggregates as a means to improve soil C sequestration.

## 5 Conclusions

Application of biochar alone did not affect aggregate stability of the humid Ultisol within two years under conventional hand-hoe tillage practice. However, when applied together with easily mineralizable *T. diversifolia* at a rate of 2.5 t ha^−1^, it increased aggregate proportion of medium-sized soil aggregates and resulted in increased SOM in the micro-aggregates. Mineral fertilizer reduced macro-aggregate stability at least in the short term, but SOC increased in the micro-aggregates. This may result in increased soil stability in the long term. We did not find a relationship between soil aggregation and soil respiration but biomass was positively related to the MWD as an indicator of soil aggregate stability. This indicates that OM input by plants is an important feedback mechanism for soil aggregation. The pattern observed in our data suggests that within the timeframe of the study, biochar is stored as free particulate OC in the micro-fraction. This shows a tendency to shift native SOC from the larger-size aggregates to the smaller-sized fraction in the short-term (2 years). Therefore, applying easily mineralizable organic matter such as *T. diversifolia* green manure may hasten build-up of macro-aggregates in the long term but this needs further investigation.

## Figures and Tables

**Table 1:** Physical-chemical properties of the soil at start of the experiment and the amendments used in the field trial (nd = not determined).

Biochar and soil			Green manure (*T. diversifolia*)	
Property	Biochar	Soil	Property	
C (g kg^−1^)	868	23.3	N (mg g^−1^)	21.5
N (g kg^−1^)	27	21.0	P (mg g^−1^)	2.3
pH	6.31	6.01	K (mg g^−1^)	43.2
EC (S mm^−1^)	196	88.0	Ca (mg g^−1^)	13.6
K (mg kg^−1^)	1490	223	Mg (mg g^−1^)	2.6
Ca (mg kg^−1^)	1920	1950	S (mg g^−1^)	2.5
Mg (mg kg^−1^)	150	312	Mn (mg kg^−1^)	264
Mn (mg kg^−1^)	188	782	B (mg kg^−1^)	53.2
S (mg kg^−1^)	36.5	14.0	Zn (mg kg^−1^)	89.7
Cu (mg kg^−1^)	0.77	1.97	Mo (mg kg^−1^)	1.29
B (mg kg^−1^)	1.07	0.33	Fe (mg kg^−1^)	951
Zn (mg kg^−1^)	108	13.5	Cu (mg kg^−1^)	11.0
Na (mg kg^−1^)	180	15.9	Na (mg kg^−1^)	72.7
Fe (mg kg^−1^)	164	67.2		
P (mg kg^−1^)	135	9.30		
Al (mg kg^−1^)	559	939		
C.E.C (meq 100 g^−1^)	18.2	16.2		
Silt (%)	nd	17.5		
Sand (%)	nd	10.7		
Clay (%)	nd	71.6		

**Table 2:** Experimental treatments for determining the effect of Biochar, T. diversifolia green manure and Urea on soil aggregate distribution and C content in a maize field in western Kenya.

Treatment	Biochar		Green manure (*T. diversifolia*)		Mineral N fertilizer (Urea)	
	Rate (t ha^−1^)^a^	*Code*	Rate (t ha^−1^)^b^	Code	Rate (kg N ha^−1^)^c^	Code
1 (B_0_T_0_U_0_)(Control)	0.0	B0	0.0	T0	0.0	U0
2 (B_0_T_2.5_U_0_)	0.0	B0	2.5	T2.5	0.0	U0
3 (B_0_T_5_U_0_)	0.0	B0	5.0	T5	0.0	U0
4 (B_0_T_0_U_120_)	0.0	B0	0.0	T0	120	U120
5 (B_0_T_2.5_U_120_)	0.0	B0	2.5	T2.5	120	U120
6 (B_0_T_5_U_120_)	0.0	B0	5.0	T5	120	U120
7 (B_2.5_T_0_U_0_)	2.5	B2.5	0.0	T0	0.0	U0
8 (B_2.5_T_2.5_U_0_)	2.5	B2.5	2.5	T2.5	0.0	U0
9 (B_2.5_T_5_U_0_)	2.5	B2.5	5.0	T5	0.0	U0
10 (B_2.5_T_0_U_120_)	2.5	B2.5	0.0	T0	120	U120
11 (B_2.5_T_2.5_U_120_)	2.5	B2.5	2.5	T2.5	120	U120
12 (B_2.5_T_5_U_120_)	2.5	B2.5	5.0	T5	120	U120

a One kg of biochar per treated plot.

b 1 and 2 kg of *T. diversifolia*, respectively. Biochar C = 86.8%, *T. diversifolia* C ∼48%.

c 100 g per treated plot.

**Table 3:** Variance analyses of effects of biochar, T. diversifolia and urea and their interactive effects on soil aggregate properties (LM large macro-aggregates; SM small macro-aggregates; M micro-aggregates; S + C silt and clay).

Factor	LM		SM		M		S + C		Total C	
	F	P	F	P	F	P	F	P	F	P
B	0.06	0.805	0.0	0.946	1.0	0.332	0.0	0.974	12.31	**0.001**^a^
T	0.25	0.782	0.2	0.847	0.68	0.520	9.1	**0.002**^a^	1.75	0.176
U	0.04	0.853	10.2	**0.005**^a^	0.12	0.734	31.5	**0.000**^a^	2.84	0.093
B × T	1.89	0.178	0.0	0.960	0.69	0.513	1.6	0.225	0.16	0.856
B × U	0.00	0.990	0.2	0.682	0.88	0.360	0.0	0.848	0.17	0.679
T × U	0.43	0.659	1.0	0.404	0.59	0.568	9.0	**0.002**^a^	0.08	0.926
B × T × U	1.85	0.184	5.0	**0.019**^a^	7.1	0.006^*^	17.0	**0.000**^a^	0.19	0.827

a Values in bold are statistically significant.

**Table 4:** Content of SOC (g kg^−1^ soil) associated with different soil fractions. SE = standard Error, n = 3 (LM large macro-aggregates; SM small macro-aggregates; M micro-aggregates; S + C silt and clay).

Treatment ID	LM		SM		M		S + C		TOC	
	Mean	SE	Mean	SE	Mean	SE	Mean	SE	Mean	SE
1 (B_0_T_0_U_0_)(Control)	27.4	(±0.24)a	25.5	(±0.46)ab	26.4	(±1.18)c	31.6	(±0.48)c	29.5	(±0.18)a
2 (B_0_T_2.5_U_0_)	25.7	(±0.67)b	25.3	(±0.43b	28.5	(±0.86)a	32.4	(±0.84)ab	28.2	(±0.88)b
3 (B_0_T_5_U_0_)	25.7	(±0.81)b	25.6	(±0.75)ab	27.2	(±1.06)b	33.6	(±1.49)a	27.8	(±0.65)b
4 (B_0_T_0_U_120_)	25.9	(±0.60)b	23.6	(±1.22)c	27.9	(±0.58)ab	33.3	(±0.69)a	27.7	(±0.06)b
5 (B_0_T_2.5_U_120_)	24.9	(±0.77)c	26.0	(±0.48)a	26.4	(±0.85)c	31.9	(±1.24)c	27.3	(±0.56)b
6 (B_0_T_5_U_120_)	25.3	(±1.34)a	24.8	(±1.56)bc	27.7	(±1.79)ab	32.1	(±1.92)c	27.5	(±0.66)b
7 (B_2.5_T_0_U_0_)	24.3	(±0.68)cd	24.6	(±1.44)bc	27.0	(±1.98)c	32.5	(±1.91)ab	27.1	(±0.90)bc
8 (B_2.5_T_2.5_U_0_)	24.2	(±1.17)cd	23.9	(±1.11)c	26.8	(±1.32)c	31.7	(±3.84)d	25.9	(±0.13)c
9 (B_2.5_T_5_U_0_)	24.8	(±1.17)c	23.8	(±1.08)c	26.5	(±0.38)c	31.3	(±1.03)d	26.6	(±0.65)c
10 (B_2.5_T_0_U_120_)	24.2	(±0.78)cd	24.1	(±1.44)c	25.0	(±0.75)d	30.8	(±1.24)cd	26.0	(±0.61)c
11 (B_2.5_T_2.5_U_120_)	22.5	(±1.41)e	23.8	(±0.84)c	25.3	(±1.13)d	32.5	(±0.52)b	25.8	(±0.01)c
12 (B_2.5_T_5_U_120_)	23.9	(±1.27)e	24.1	(±0.72)c	25.4	(±0.90)d	32.6	(±0.58)b	26.3	(±0.81)c

In each column, means with the same letter are not significantly different at p < 0.05.
